# On the Etiology of Epidemic Cholera

**Published:** 1833-08

**Authors:** Alexander Turnbull Christie

**Affiliations:** the Honourable East-India Company's Service; Member of the Royal Asiatic Society of Great Britain and Ireland, of the Wernerian and Royal Medical Societies of Edinburgh, &c.


### THE EPIDEMIC CHOLERA.

*On the Etiology of Epidemic Cholera.* By ALEXANDER TURNBULL CHRISTIE, M.D., of the Honourable East-India Company's Service; Member of the Royal Asiatic Society of Great Britain and Ireland, of the Wernerian and Royal Medical Societies of Edinburgh, &c.

THERE has been always much difference of opinion respecting the nature of those morbid conditions to which all the symptoms of cholera are referrible; or, in other words, of its proximate cause. This was to be expected, so long as we continued in ignorance of the morbid changes which accompany, or form part of it; but now that we are in possession of a great mass of well-established facts, relating both to its autopsy and symptoms, we may proceed with more confidence to trace its causes, and to determine whether it is subject to any uniform laws.

Overlooking those theories, or rather hypotheses, which attribute all the phenomena of cholera to an immaterial something,* to some peculiar and undefined influence of electricity in the blood and textures of the body, to concussion of the brain,† to torpor of the extreme vessels of the liver, or to some other equally obscure cause; let us proceed to examine the more prevalent opinions of the day. These refer the disease to diminished nervous energy; to congestion of the veins of the viscera; to inflammation of the brain, of the spinal marrow, or of some portions of the mucous membranes; or to the altered state of the blood.

To say that any disease is owing to diminished nervous energy, is, I conceive, only using a peculiar phraseology, instead of furnishing a scientific explanation of symptoms. No one will say that he knows any thing of diminished nervous energy except by its effects. Many however conclude, from

* Bombay Report. † Kennedy on Cholera.

the existence of certain phenomena, that there is such a thing as diminished nervous energy; and the next moment they turn round, and have recourse to it, for the explanation of the very phenomena from which its existence was at first deduced.

But it appears to me that we can with less propriety attribute cholera to diminished energy of the nervous system, than almost any other disease with which we are acquainted; for, in cholera, the external senses, the voluntary, and some involuntary motions, continue, in a large proportion of cases, unimpaired; and one of the most important and extensive secretions, viz. that of the mucous membranes, far from being diminished, is always prodigiously increased. Even the other secretions are not invariably diminished; and, when they are so, we can easily perceive that it does not arise from any defect of the nervous system, but from a deficiency of the circulating fluid, which has been withdrawn from these organs, and determined towards the mucous membranes, the seat of increased action.

Cholera is generally described as being accompanied by great debility of the circulating system. This, I suspect, is not strictly correct; for, although a small pulse is almost an invariable symptom of the disease, it is by no means a proof of arterial debility; but merely that the quantity of blood circulating through the vessel has been diminished. It is thus conceivable how an artery, though containing a smaller quantity of blood than usual, may have its action actually increased. Were the smallness of the pulse owing to general debility of the circulating system, we might expect it to arise more gradually, and to continue longer after the removal of the disease, than we find to be the case in cholera. A person enjoying good health is attacked with the disease: his pulse becomes rapidly smaller; his features collapse from diminution of blood; and, if the disease prove fatal, the blood is found accumulated in the internal vessels. Should he recover, the pulse immediately regains its fulness, and sometimes has its action much increased. These facts naturally lead to the conclusion, that the smallness of the pulse is not owing to debility; but only to the circumstance of the blood having been withdrawn from the surface, and determined to the interior.

How the diminished nervous energy produces increased secretion from the mucous membranes, congestion, pain in the abdomen, spasms, &c. we are not told; one of the advocates* of the hypothesis merely says, "that the depravation of ner-

* Mr. Orton.

vous influence thus produced extends to all the functions, and immediately produces the disease." Well may we say with the reviewer, in the Edinburgh Medical Journal, that "this is a complete assumption, unsupported by any proof, evidence, or any plausible pretext whatever." I have thought it necessary to dwell thus long upon this hypothesis, since, if adopted, it might be liable to lead to an injurious mode of practice. One of the most powerful and universal remedies in the disease is bloodletting; but it is difficult to understand upon what ground it could be recommended in a malady depending upon diminished energy of the nervous system.

The theory which refers all the phenomena of cholera to venous congestion, is much more entitled to attention than that we have just been considering. Venous congestion is present in all severe and fatal cases of the epidemic; but this alone is not a proof of its being the proximate cause. By the term proximate cause, we mean that morbid condition without which the disease could not exist; which, in fact, constitutes the disease, and to which all the other morbid changes, as well as the symptoms, are referrible. It necessarily follows that it must not only be of invariable occurrence, but it must always precede all the symptoms; must form the very first link in the chain of morbid phenomena which accompany the disease, and must be proved to be an adequate cause of these phenomena. Does the venous congestion in cholera answer all these conditions? I will shew that it does not. In a great proportion of cases, the first symptoms are vomiting and purging, with a sense of uneasiness at the epigastrium; and these often continue for a long time, before any others make their appearance. It often happens that a patient ejects a large quantity of serous fluid, by vomiting and stool, when, at the same time, his circulation is not much disturbed, and there are no other symptoms (except perhaps anxiety) to indicate the presence of cholera. No one, however, doubts of the identity of the disease; and the practitioner does not hesitate to treat it *secundum artem*. Now, in such a case, there is surely no indication of venous congestion, and it therefore cannot be the first morbid change in the disease. So little appearance of congestion is there in the very first stage of many cases of cholera, that the pulse is then full and strong; whereas, had the blood left the surface to be accumulated in the internal parts, the pulse must necessarily have been small and weak. But we can produce an artificial case of cholera, which differs from the epidemic only in degree, and we can thus carefully study its progress, and the order of its phenomena. A drastic purge acts upon the gastro-enteric mucous membrane, occasioning an increased secretion; nausea, and uneasiness in the bowels, are produced; abundant dejections are expelled, accompanied sometimes by vomiting, and the blood, at length, retires from the surface towards the viscera, until the operation of the medicine ceases. No one will, surely, pretend that the first effect of the physic is to withdraw the blood from the surface, in order to supply material for the flow of excretions that are afterwards to take place; besides, such an absurdity is, as we have already stated, exactly the reverse of the order of the phenomena. The same reasoning applies to the true cholera. But, allowing for a moment venous congestion to be the proximate cause, let us inquire how it would account for the morbid appearances, and the symptoms of the disease. A great accumulation of blood in the viscera of the abdomen may be supposed capable of forcing out, as it were, a large quantity of fluid into the alimentary canal; but, if such were its mode of action, why are all the constituent parts of the blood not forced out? why is the colouring matter left behind? why is the constitution of the cholera secretion different from that of the blood? But, if this difficulty occurs in regard to the secretions of the stomach and intestines, how much greater is the difficulty which presents itself respecting the cause of that thrown out from the inner surface of the bladder, where there is seldom any congestion, and from the surface of the eyes, and the lining membrane of the mouth, which are far from the seat of the cause to which they are supposed to be owing. Lastly, this theory affords us no reason for the perspiration, which is a very common symptom; on the other hand, it withdraws the circulating fluid from the surface, in order to supply a *cause* for the increased secretion from the gastro-enteric mucous membrane, and thus leaves no cause whatever for the secretion of the skin.

In regard to the theory which attributes the proximate cause to inflammation, suffice it to say, that inflammation in any part of the body is of very rare occurrence, in cases of the epidemic.

The altered condition of the blood has sometimes been supposed to be the proximate cause of the disease. But it often does not occur until the other symptoms have been fully developed, and therefore does not answer one of the conditions of a proximate cause; and it is sometimes met with in other complaints, when it is not accompanied by any symptoms similar to those of cholera. I do not believe that the changes of the blood arise entirely from deficient oxygenation; for the effects of unoxygenated blood are very different from any thing we observe in cholera. Mr. Brodie says, that "dark-coloured (unoxygenated) blood, which has been transmitted through the circulating system during the suspension of respiration, would seem to act like a narcotic poison upon the brain. *No sooner does it enter that organ, than deleterious effects are immediately produced;* the animal falls into a state of stupor, the pupils of the eyes become dilated, the respiration is laborious, the muscles of the body are convulsed, and the animal dies poisoned by its own blood." Very different is the case in cholera; for the patient often continues perfectly sensible, his breathing free, and his muscles obedient to his will, for *many hours,* during which dark-coloured blood has circulated through his veins and arteries. Although the blood is variously altered, and darker than usual, yet I doubt much whether these changes be owing merely to imperfect decarbonization of the circulating fluid. The only reason we have for apprehending such to be the case, is the statement of Dr. Davy, who says that "the air expired from the lungs of the sick with cholera did not contain more than one third of the carbonic acid gas contained in the breath of healthy people." But we are not told by what experiments this was ascertained, and whether it was the result of extensive observation; and, besides, greater slowness of the circulation may have occasioned this deficiency to a certain extent; although the blood which actually passed through the lungs was perfectly decarbonized. The dark colour of the blood, therefore, was probably owing to some other cause than to its not having been freed from its carbon; and, upon any supposition, it could not serve to explain the different symptoms of the disease.

It is much to be wondered at that, in all the observations that have been published on the pathology of cholera, so little weight has been given to the disordered condition of the mucous membranes, which forms an invariable character of the disease. This appears the more surprising, when we consider the importance of the mucous membranes in the animal economy; their very great extent; their great sensibility; the numerous sympathies that exist between them and every other part of the body; the many instances in which their diseases have become epidemic; and the invariable derangement of all, or at least of some portions of them, in cholera.

Although many medical men have accurately described the various appearances of the secretion thrown out by the stomach and intestines in cholera, they have very seldom extended their observations to the diseased action of the gastro-enteric mucous membrane, by which these secretions are produced. A very common, and perhaps a natural prejudice, inclines us to consider the intestinal tube merely as a conduit for the passage of excremential and peccant matters, and that all kinds of fluxes are only efforts of this tube to rid the bowels of diseased substances, the presence of which is prejudicial to health. Farther, there appears to be a disinclination, with many pathologists, to admit that the gastro-enteric mucous membrane is ever the seat of the pathological cause of a disease. Hence it is that cholera morbus has been referred to the irritation of diseased bile, diarrhœa to diseased matters irritating the intestines, and Indian cholera and dysentery to disordered states of the liver, of the brain, nervous system, &c. It is certain that cholera was not considered to be a disease *sui generis* until it occurred as an epidemic. Previous to that period it was generally denominated flux; and, by the natives of India, it is simply denominated vomiting and purging. Under these names, it is a disease that has been well known at very distant periods and places. But, as soon as it became epidemic, its name was altered, and its nature confounded. The discovery of its pathological cause was considered by many to be impossible; and those who did theorize upon it referred it to almost every diseased condition of the human frame, except that to which it had formerly and frequently been attributed.

I have already demonstrated, on another occasion, that mucous membranes are liable to two distinct morbid affections, viz. inflammation and catarrh; and that either of these may occur alone, or conjoined with the other. Without entering very fully into the views respecting the pathology of mucous membranes, which I have explained in the work alluded to, and which I believe have been very generally adopted, it will be necessary to give some explanation of them, in order to make their application to the pathology of cholera properly understood.

It has been stated as a general law, by several eminent authors, that inflammation of mucous membranes is accompanied by increased mucous secretion; and pathologists almost invariably attribute catarrh to an inflammation of the mucous membrane in which it occurs.* This, I apprehend, is far from being correct; for are there not numerous examples of inflammation of a mucous membrane without increased secretion, and of catarrh without inflammation? We have examples of the former in ophthalmia, inflammatory sore throats,

* This is the opinion of Laennec. Vide De l'Auscultation Médiate, t. ii. p. 65. It also appears to be Broussais' opinion.

some cases of gastritis, and perhaps also of enteritis: of the latter, in a few cases of common catarrh, in diarrhœa, and also (as I shall have occasion to shew in a future part of this Essay) in Indian cholera.

Acute bronchitis frequently affords an example of simple inflammation of a mucous membrane, without increased secretion. In some cases of it, the secretions during the first stage are diminished; and then an increased secretion from the inflamed membrane always relieves the disease.* In the first stage of gastritis, and of enteritis, the secretions of the gastro-enteric mucous membrane are generally diminished; and these diseases also are relieved by an increased flow of the natural secretions of the membranes.

There are also numerous examples of catarrh without inflammation. Common catarrh of the air-passages, without inflammation, is familiar to every one. Diarrhœa generally does not exhibit the slightest symptom of inflammation; and in it the secretion of the enteric mucous membrane is increased and vitiated.

There is a species of diarrhœa endemical in some parts of the Southern Mahratta country, the symptoms of which are copious and frequent dejections of a serous fluid, a small pulse, and sometimes coldness of the skin, without the slightest symptom of inflammation. When it is of long continuance, however, inflammation generally supervenes; and the enteric mucous membrane, when examined after death, presents various diseased appearances. But, in one case in which it had existed for a very long time, and at length proved fatal, the mucous membrane of the alimentary canal had a blanched appearance, with only a few rose-coloured spots near the pylorus, and in the large intestines. It is most probable that these spots were not inflammatory, but what Billard calls "rougeur pointillée non inflammatoire." At all events, they were not of a magnitude that could account for the violent and long continued catarrhal affection that affected the patient.

There are some very valuable observations on this subject in M. Billard's work "De la Membrane Muqueuse Gastro-Intestinale," at page 358. He mentions that it is by no means uncommon to meet with cases, in which there are very abundant dejections of serous fluids, and in which no trace of inflammation can be detected in the mucous membrane of the primæ viæ after death. Among others, he describes the case of a priest, who was attacked with an intestinal flux, and passed

* Vide Hastings' Treatise on Inflammation of the Mucous Membrane of the Lungs, p. 161.

different vitiated humours, for the space of thirteen days. He then died; and, upon examining his corpse, no appearance of inflammation was found in any part of the intestinal canal. He farther states, that the same thing is daily met with in the theatres of anatomy; and therefore justly concludes, that these fluid excretions from the bowels cannot be considered as indications of inflammation of the mucous membrane of the intestines.

In the description of the symptoms and of the autopsy of the epidemic cholera, it has been mentioned that there is seldom the slightest appearance of inflammation; that the most prominent symptoms are copious vomiting and purging of a sero-fibrinous secretion; that no traces of inflammation could be detected in the mucous membranes after death; but that these membranes, the pulmonary and urinary, as well as the gastro-enteric, were all whiter than natural, and lined with a fibrinous secretion.

Let us now consider the effects of these two morbid affections, viz. of inflammation and catarrh, on the general system. These effects on the circulation are very different, and in fact completely opposite, acute inflammation being accompanied by increased action; whereas, in slight cases of catarrh of any portion of the mucous membranes, the circulation is scarcely, if at all, affected; and, in severe cases, both the size of the pulse and heat of the skin are diminished. This is so important in enabling us to explain the etiology of various diseases, that it will be necessary to shew that numerous proofs are not wanting to establish it as a general law.

It has frequently been observed, that simple diarrhœa, which is merely a catarrh of the mucous membrane of the large intestines, is accompanied by a weak small pulse, and a sense of coldness.

A powerful dose of physic, occasioning an abundant secretion from the bowels, is frequently followed by a small pulse, and coldness of the skin. This was illustrated in a most striking manner in the case of one of my own servants, a young Mahomedan, who had taken too large a dose of the croton tiglium, which occasioned hypercatharsis. His evacuations, after a time, consisted only of mucus and serum; his pulse was scarcely perceptible at the wrist; his extremities were cold, and his features contracted. He was in this state when I first saw him; and from all these symptoms I immediately concluded that he had an attack of the cholera. When I learned the true nature of his complaint, I gave him sixty drops of laudanum, and he soon recovered.

Simple catarrh of the nose, fauces, and air-passages, is always attended by chilliness and a diminution in the size of the pulse; but the influenza of 1803, which was a severe catarrh with inflammation, was denominated a fever on account of the increased action of the heart and arteries which characterized it.

These proofs, in support of the principle, that catarrh of any part of the mucous membranes occasions a diminution in the size of the pulse, and heat of the skin, will at present suffice. Many of a similar nature will doubtless suggest themselves to my readers; and I will now endeavour to trace the cause of the phenomenon.

As it is of importance, for the proper understanding of this part of our subject, to have clear notions of the power excited by the capillaries in the circulation of the blood, I will quote the following observations from the elegant work of Dr. Arnott on Natural Philosophy:

"We have seen (says he) that the heart keeps up a tension or pressure in the arteries, of about four pounds on the square inch of their surface, and with this force, therefore, is propelling the blood into the capillaries. If these last were passive tubes, constantly open, such force would be sufficient to press the blood through them with a certain uniform velocity; but they are vessels of great and varying activity: it is among them that the nutrition of the different textures of the body takes place, as of muscle, bone, membrane, &c.; and that all the secretions from the blood are performed, as of bile, gastric juice or saliva, &c.; and, to perform such varied and often fluctuating offices, they require to be able to control, in all ways, the motion of the blood passing through them. The capillaries of the cheek, under the influence of shame, dilate instantly, and admit more blood, producing what is called a blush; under the influence of anger or fear, they suddenly empty themselves, and the countenance becomes pallid; tears or saliva, under certain circumstances, gush in a moment, and in a moment are again dried up: if a person having inflammation in one hand be blooded from corresponding veins in both arms at the same time, twice or thrice as much blood will flow from the diseased side as from the other. Similar changes occur in many other instances. Now, the only mechanical action of vessels, capable of causing these phenomena, must occur in contractile or muscular coats; and, with reference to such action, it merits notice that arterial branches have always more of the fibrous or contractile coat in proportion as they are smaller.

"A muscular capillary tube strong enough to shut itself against the arterial current from the heart, is also strong enough to propel the blood to the heart again through the veins, even if the resistance on that side were as great as the force on the other. * * *

"It is capillary action which absorbs and moves the fluids of the classes of animals which have no heart. It must also be the power which moves the blood in warm-blooded monsters formed without hearts. There are cases of apparent death among human beings, where the heart remains inactive for days, and yet a degree of circulation sufficient to preserve life is carried on by the capillaries. In illustration of capillary action, we have also the absorption, by the lacteals, of nutriment by the alimentary canal; and perhaps, to a certain extent, the circulation of the blood in the livers of animals. In this last case, the blood collected by veins from the abdominal viscera, instead of going directly to the heart, is again distributed through the liver by the branches of the vena portæ, and is then again collected by ordinary veins, and carried to the heart: it thus moves through two sets of capillaries in passing from the arteries to the heart again.

The action of the capillaries is the cause of that singular phenomenon which prevented the ancients from discovering the circulation of the blood, viz. the empty state of the arteries after death. All the muscular parts of an animal, including therefore the contractile coats of vessels, retain their life, or power of contracting, for a considerable time after respiration has ceased, as is seen in the recovery of persons apparently drowned or suffocated; in the leaping of a heart taken from an animal just killed; in the actions resembling life which can be produced, by the agency of galvanism, in a body recently dead; but the fact is seen still more aptly for our purpose, in the total disappearance of a local inflammation after the death of the patient; for inflammation involves a gorging, or over-distention of the capillaries, into which, when the heart has ceased to press blood, the contractile force remaining in them, even under disease and in a dead animal, is sufficient to squeeze the blood out of them, and often to remove all trace of the malady which has killed. In ordinary cases, then, the capillaries throughout the body remain alive and active for a considerable time after breathing has ceased, working like innumerable little pumps, and emptying the arteries into the veins. As the red blood is their proper sustenance as well as stimulus, they work as long as there is any of it coming from the arteries behind them; except, however, the capillaries of the lungs, which soon cease to act, because, after breathing has ceased, they are filled with black blood, and are moreover compressed by the collapse of the chest; and all the blood accumulates behind them. The capillaries may continue to be filled from the arteries, either in consequence of their elasticity opening them with what is called a suction power, or of an absorbent power dependent on life, like that of the lacteals and of the absorbents all over the body, and perhaps of the vessels in the roots of vegetables. When death is produced by lightning, or by the poisons which destroy all muscular irritability, the arteries after death are found to contain blood like the veins. In a living body, if an artery be tied, the part beyond the ligature is soon emptied into the veins, and becomes flat. The experiment has been made even upon the aorta itself."

It is an important law of the animal economy, that there is always a determination of blood towards a part whose action is increased. Let us suppose then the action of the minute vessels of any part to be increased in consequence of inflammation, a determination of blood will take place towards them; but, as the seat of this affection is in the capillaries which connect the arteries with the veins, it is evident that there can be no outlet for the unusual quantity of blood they now receive; it must therefore be propelled forwards with a heightened momentum, which, upon reaching the heart, will be communicated by it to every part and of the sanguiferous system, producing the quick and hard pulse, and hot skin, which accompany all severe inflammations. But very different effects must arise from the increased action of secretory vessels; for, since they discharge their contents upon open surfaces, the more powerfully they act, the greater will be the quantity of fluid withdrawn from the circulation; and the total quantity of it will consequently be diminished. Instead of general excitement, therefore, of the circulating system, as in inflammation, there must be symptoms of diminished action, or, to speak more correctly, the size of the pulse towards the surface will be lessened, from the arteries containing a smaller quantity of blood than usual. Let us suppose, farther, that the excretory vessels of the gastro-enteric mucous membrane have their action increased, or, in other words, are affected with catarrh, the current of the blood will be directed towards this membrane, to supply the expenditure, less will consequently be sent to the other parts of the body; the heart and large blood-vessels having the bulk of their contents in this way diminished, will contract with less effect, and their action will become languid. But we can scarcely suppose that in all cases the exact measure of blood required for the supply of the discharge will be directed towards the affected parts; and if too little be afforded, the catarrh will be of short duration; if too much, there will be congestion. The latter is likely to occur in almost all severe cases; for, an impulse having been given to the flow of the blood towards the alimentary canal, more of it will probably be thus carried to the parts than is precisely equal to supply the increased secretion; and the surplus will therefore accumulate in the veins of the viscera. This state of things will continue until the unnatural drain, which had thus diverted the stream of the vital fluid from its usual course, is stopped, and the circulation, again set free, is enabled to distribute in due proportions its supplies to the remotest parts of the system. Thus we find that inflammation and catarrh are exactly opposed to each other; and, accordingly, many remedies which are employed against the former act by producing increased secretion, or a catarrh in the mucous membrane; many which are found useful in the cure of the latter occasion an inflammatory action. Of the former we have a familiar instance in inflammatory sore throat, which is relieved by an increased secretion from the mucous membrane of the affected parts; the latter is equally evident in the beneficial effects derived from the employment of opium and stimulants in the catarrhal cholera.

One reason, probably, why an accurate distinction has not hitherto been made between inflammation and catarrh, is that the former seldom continues for any length of time in a mucous membrane without exciting the latter, and *vice versa*. When they occur together, the state of the circulation will of course be modified according to circumstances: it will be increased, if the inflammation predominate; diminished towards the surface, if the catarrh predominate. Accordingly, we find that, in some cases of dysentery, the pulse continues tolerably full at the wrist, and the skin hot, while, in other cases, the pulse is nearly imperceptible, and the skin cold; the former being those in which the inflammation, the latter those in which the secretion, is greater.

From numerous experiments I performed upon dogs, for the purpose of ascertaining the mode of action of several of the more active medicines on the gastro-enteric mucous membrane, and which have been detailed in another work, I found that certain medicines, especially in large doses, increased the secretion of this membrane, at the same time rendering it white; and that others caused an inflammatory action. I also ascertained that if a stimulating medicine be made to act for a length of time upon one spot, it causes inflammation; whereas a short continuance of its action produces only increased secretion. In this way I observed that large doses of tartrate of antimony, of muriate of mercury, and of calomel, produced a great secretion from the gastro-enteric mucous membrane, and at the same time rendered it white; opium occasioned a red colour; and in some cases in which calomel had remained a long time in the stomach, it adhered to its inner surface, and produced patches of inflammation.

In June 1826, I gave a scruple of tartrate of antimony to a patient labouring under an acute pulmonic affection. In half an hour it caused him to vomit a large quantity of serous fluid; shortly afterwards he vomited three yards of a tape-worm, and, in the course of two or three hours, it produced several copious and perfectly watery stools. The size of his pulse was much reduced; his skin became cool and slightly moist, and in the course of twelve hours all these symptoms went off, and left him quite well. I afterwards gave similar doses of the medicine to three or four other patients, and in these it invariably produced watery vomiting and purging, coldness of the skin, a small pulse, and general debility.

It would appear, then, that a scruple of tartrate of antimony powerfully increases the secretion of the gastro-enteric mucous membrane, without at the same time inducing an inflammatory action. It consequently determines the blood towards the abdominal viscera, and thus reduces the size of the pulse and the heat of the skin. These effects it produced so powerfully, in one of the cases above alluded to, that a person unacquainted with the circumstances might have almost mistaken it for a case of cholera. This practice, I should think, cannot be always unattended with danger; for, were the medicine, instead of quickly passing through the alimentary canal, (and thus merely exciting the action of the excretory vessels,) to lodge in any part of it, there can be no doubt that its continued action would excite inflammation.

The above facts shew that the tartrate of antimony has not a direct sedative property, as some maintain: on the contrary, it powerfully excites the secretion of the gastro-enteric mucous membrane; and the small pulse, cold skin, and debility, are only secondary effects, which go off as soon as the increased secretion is checked.

The experiments of Brodie* and Magendie,† and the observations of various other writers,‡ prove that tartrate of antimony frequently produces inflammation of the gastro-enteric mucous membrane. In the experiments of Magendie, in which the inflammation was produced by tartrate of antimony, the œsophagus was tied, so as to prevent vomiting; which shews

* Vide Phil. Trans. for 1812, vol. 102, p. 205.
† Vide Orfila, Traité des Poisons, third edit. p. 458; also 479 et seq.
‡ Vide Billard, De la Membrane Muqueuse, p. 201 et seq.

that the inflammation was produced by the continued action of the medicine on the mucous membrane. Other experiments are related by Brodie, in which large quantities of tartrate of antimony given to dogs produced no inflammation whatever.*

If my reader has accompanied me attentively through the preceding (I am afraid rather tedious) investigation, I feel confident he will be prepared to admit the following conclusions.

1. Mucous membranes are liable to two distinct simple morbid affections; viz. inflammation and catarrh.

2. Catarrh consists of a diseased action of the secretory apparatus of a mucous membrane, which produces an increased and vitiated secretion, and is characterized by the membrane in which it occurs being generally whiter than natural, and by the quantity of the blood towards the surface of the body being diminished.

3. Either of these morbid affections may occur alone in a mucous membrane, or conjoined with the other.

It is worthy of remark, that almost all the remedies which have been found successful in the treatment of the epidemic cholera, are such as stimulate the arterial capillaries, and therefore promote the circulation of the blood; for we find, by the observations of Dr. Arnott, that these minute vessels have as much share in the circulation as the heart itself. Blisters, sinapisms, frictions, and heats, are applied to the surface; stimulating mixtures, laudanum, spices, ammonia, are given internally, all with the same view of exciting the action of the capillaries, and thus causing them to restore the balance of the circulation. It is not improbable that the action of the arterial capillaries, as well as of the heart, may be diminished by the altered state of the blood having deprived them of their natural stimulus; but that they are not *positively debilitated* has been already pointed out.

Let us now apply these principles to the explanation of the phenomena of the epidemic cholera. It has been already mentioned that the first link in the chain of morbid phenomena, in cholera, is a disordered state of the gastro-enteric mucous membrane, which occasions an increased excretion, or catarrh. As in all other similar affections, whether spontaneous or excited by artificial means, a determination of blood takes place towards the parts, the quantity of blood towards the surface is diminished; and, when the catarrh is sudden,

* The preceding observations enable us to understand why some purgatives are said to be warm, and others cold; the former being such as simply stimulate the bowels, and excite their peristaltic motion; the latter those which occasion an increased secretion from their mucous membrane.

severe, and extends to all the mucous membranes, it constitutes a powerful cause fully equal to produce collapse, and all the worst symptoms of the disease.

As it is of importance to demonstrate positively that the catarrhal affection always precedes all the other morbid phenomena in the disease, it will be necessary to enter a little into detail upon this point. It has already been shewn repeatedly that a disordered state of the bowels, very much resembling cholera, has frequently arisen from a powerful dose of physic; and, during the prevalence of the epidemic, a strong dose of a saline purgative has been often seen to act as an exciting cause. But the immediate operation of purgatives is to excite the secretion of the mucous membrane of the primæ viæ, which must, in such cases, therefore have been the first deviation from healthy action that took place. Moreover, cholera often commences with an insidious diarrhœa, without any other symptom. Can there be stronger proofs than these, that the first link in the chain of morbid phenomena is a catarrh of the gastro-enteric mucous membrane?

During the prevalence of cholera at Darwar, in 1826, a sepoy of the 5th regiment of Native Infantry was brought into hospital at seven A.M. of the 10th of May. He said he had been attacked with purging early in the morning, when on guard at the jail; that the first evacuations had been natural, but that the two or three last were like rice-water, which made him apprehend that he was about to have an attack of cholera. His pulse and skin were natural; his tongue was clean; and he had no other symptom of the disease. He took fifty drops of laudanum, and a strong dose of calomel and jalap, which soon produced several healthy evacuations; in other words, restored the healthy action of the enteric mucous membrane; and he had no return of the disease.

Next morning another sepoy, who had been on guard at the jail, was brought into hospital, with frequent white, watery purging. All the other functions were natural. He took a dose of physic and some laudanum, which soon restored the healthy action of his bowels; and in the evening he was so well that he intended to return to his duty the next morning. During the night, however, or rather early in the morning, he took a large draught of cold water and some buttermilk, which occasioned a renewal of the purging. When I saw him, at six o'clock A.M., he had been purged frequently, and had vomited two or three times; his evacuations being watery, with a few white flakes. His features were considerably collapsed; his pulse at the wrist extremely small. He had already taken fifty minims of laudanum. I

immediately opened a vein in his arm, from which very dark-coloured blood flowed sluggishly, in a small stream, or in drops. The bleeding was continued until I had procured thirty ounces, when the blood became lighter coloured, his features brightened, and he expressed himself much relieved. He coughed two or three times, and appeared to breathe more freely than he had done before. The disease, however, had not been overcome; for, although his pulse had considerably improved, it was far from being of its natural fulness; he vomited once, and was purged two or three times. A strong blister was therefore applied to his abdomen, and sinapisms to his legs and feet; and during the day he took several scruple-doses of calomel, with camphor mixture and liquor ammoniæ. He slept a little during the night. Next day he had two or three feculent evacuations, and speedily recovered.

These two cases very clearly point out the nature of the disease. In the first case, and on the first day of the second case, there was evidently nothing more than catarrh of the entero-mucous membrane, and which was easily overcome by simple remedies. In the latter part of the second case there was the same catarrh of the enteric mucous membrane, but increased in severity, extending to the gastric membrane, occasioning the characteristic symptoms of cholera, and requiring for its cure the active remedies usually employed in that disease.

The following passage, forcibly illustrating this subject, is taken from one of Mr. R. H. England's Reports to the Madras Medical Board. "I have frequently," says he, "detected sepoys round an encampment, with diarrhœa unattended with vomiting, pain, or any other unusual symptom. This state, I have the strongest proofs to convince me, was the commencement of the cholera; as a few of these cases terminated fatally, with the usual symptoms of the disease. After a diarrhœa had existed several hours, I have in many cases found it resist every kind of remedy; the purging continued; the vascular action became impaired; prostration of strength ensued, and the patient sunk without any considerable pain. I have often asked these persons why they did not apply to me earlier; and the general answer was, that, as they found no great inconvenience from the diarrhœa, they deemed it unnecessary and improper to make a report of a circumstance so apparently trifling." Mr. Orton, in his Essay on Cholera, says, "This epidemic has frequently been observed to begin with a common diarrhœa, which has gradually assumed the form of cholera, so as to occasion a great difficulty in the diagnosis in the early stages." Similar observations have been made by numerous other medical men, which shew that the term cholera is with general consent often applied to a simple catarrh of the mucous membrane of the intestines, without any other symptom whatever being present.

---

